# Operative technique and early experience for robotic-assisted laparoscopic nephroureterectomy (RALNU) using da Vinci Xi

**DOI:** 10.1186/s40064-015-1076-6

**Published:** 2015-06-27

**Authors:** Fadi Darwiche, Sanjaya Swain, George Kallingal, Sanoj Punnen, Murugesan Manoharan, Dipen J Parekh, Mark L Gonzalgo

**Affiliations:** Department of Urology, University of Miami Miller School of Medicine, 1120 NW 14th Street, CRB 1560, Miami, FL 33136 USA

**Keywords:** Nephroureterectomy, Urothelial cancer, Robotic surgery, Laparoscopy, Outcomes

## Abstract

**Purpose:**

Robotic-assisted laparoscopic nephroureterectomy (RALNU) has been previously utilized for management of upper tract urothelial carcinoma. The da Vinci Xi surgical system was released in April of 2014. We describe our operative technique and early experience for RALNU using the da Vinci Xi system highlighting unique features of this surgical platform.

**Materials and methods:**

A total of 10 patients with a diagnosis of upper tract urothelial carcinoma underwent RALNU using the da Vinci Xi system between April and November of 2014. A novel, oblique “in line” robotic trocar configuration was utilized to access the upper abdomen (nephrectomy portion) and pelvis (bladder cuff excision) without undocking. The port hopping feature of da Vinci Xi was utilized to facilitate optimal, multi-quadrant visualization during RALNU.

**Results:**

Robotic-assisted laparoscopic nephroureterectomy was successfully completed without open conversion in all 10 patients. Mean operative time was 184 min (range 140–300 min), mean estimated blood loss was 121 cc (range 60–300 cc), and mean hospital stay was 2.4 days. Final pathology demonstrated high grade urothelial carcinoma in all patients. Surgical margins were negative in all patients. No intra-operative complications were encountered. One patient developed a pulmonary embolus after being discharged. No patients required a blood transfusion. Mean patient follow-up was 130 days (range 15–210 days).

**Conclusion:**

The use of da Vinci Xi with a novel, oblique “in line” port configuration and camera port hopping technique allows for an efficient and reproducible method for RALNU without the need for repositioning the patient or the robot during surgery.

**Electronic supplementary material:**

The online version of this article (doi:10.1186/s40064-015-1076-6) contains supplementary material, which is available to authorized users.

## Background

Upper tract urothelial carcinoma (UTUC) is a rare disease, accounting for only 5% of all urothelial malignancies (Jemal et al. 2004). Classic standard management of UTUC is open nephroureterectomy with excision of a bladder cuff. Laparoscopic nephroureterectomy is an alternative to open surgery and has been shown to reduce perioperative morbidity while maintaining similar oncologic outcomes (Gill et al. 2000; Waldert et al. 2009; Ni et al. 2012). Robotic assisted laparoscopic nephroureterectomy (RALNU) is an extension of the pure laparoscopic technique taking advantage of the features of the robotic platform over those of standard laparoscopy. These enhancements include additional degrees of freedom with wristed instruments, motion scaling, and 3-dimensional visualization. These features make isolation of the distal ureter and bladder closure more feasible with the robotic surgical platform compared to pure laparoscopic surgery.

Several techniques have been described for RALNU using the da Vinci Si system including repositioning of the robot and/or patient (Hu et al. 2008) or without patient repositioning or robot redocking (Lee et al. 2013; Hemal et al. 2011; Zargar et al. 2014). In April of 2014, the da Vinci Xi surgical system was released which came with several upgrades and modifications compared to the previous Si version. Herein, we report the first description of RALNU using a novel, oblique “in line” trocar configuration and camera port hopping technique with the da Vinci Xi system.

## Methods

A retrospective analysis of our institutional review board approved robotic surgery database was performed to identify subjects who underwent RALNU. A total of ten consecutive patients with a diagnosis of upper tract urothelial carcinoma underwent RALNU using the da Vinci Xi surgical system at the University of Miami Hospital between April and November of 2014.

Preoperative variables included patient demographics [age, sex, body mass index (BMI)], side of tumor, and biopsy pathology or cytopathology results (if available). Outcome measures included specimen pathology, operative time, estimated blood loss (EBL), complications, length of hospitalization, and length of catheterization.

### General considerations

**Figure 1:**
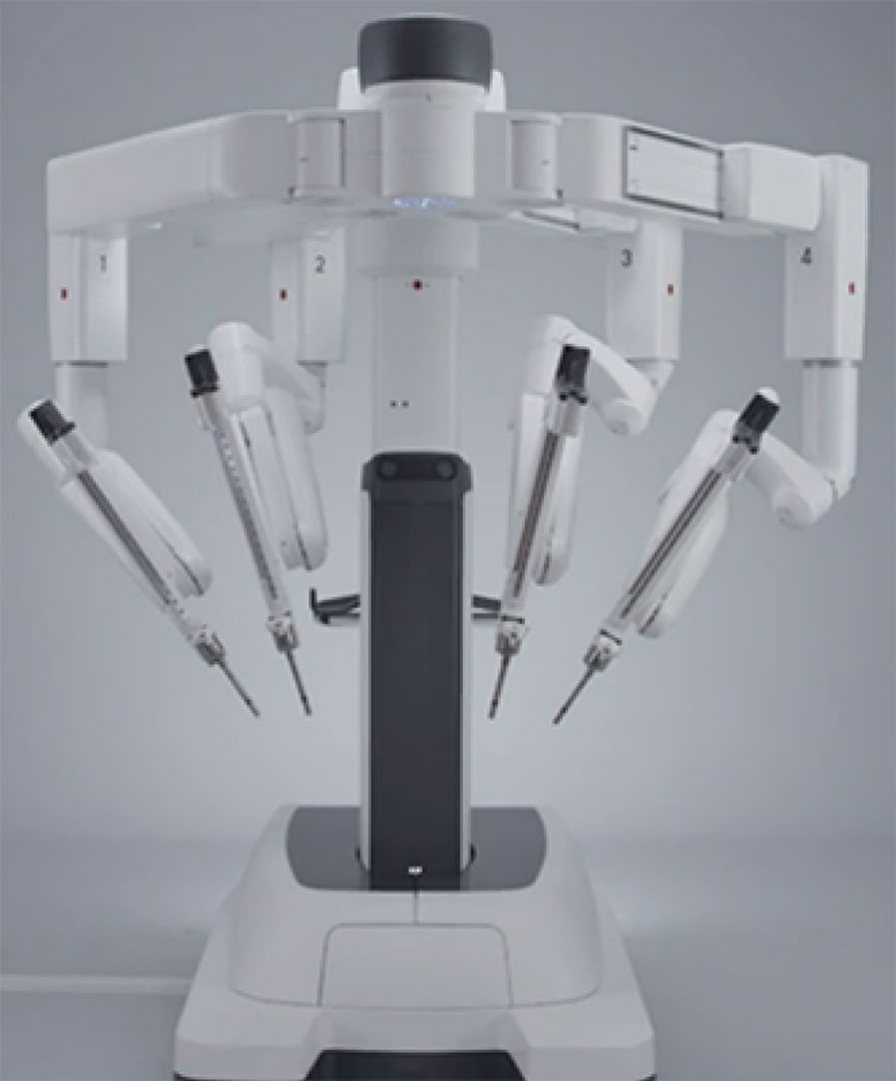
da Vinci Xi surgical system.

The da Vinci Xi surgical platform utilizes a four overhead arm architecture (see Figure 1). The robotic arms are thinner compared to prior models of da Vinci with joints that can be manipulated to provide additional patient clearance from the robotic arms. All da Vinci Xi robotic instruments have longer shafts and the endoscope has a camera built into the distal tip. The robotic camera easily fits into an 8 mm trocar which allows its insertion into any of the four robotic arms. After the trocars have been placed, the robot is positioned with the target sign from the boom aligned over the designated camera port. The camera is docked first, after which targeting of the organ being removed is performed to allow automatic adjustment of the remaining arms before coupling with trocars. A “patient clearance” button can be used to rotate joints and help keep arms away from the patient. In general, we found that maneuvering of the robot and docking was easier using the Xi system compared to prior models. The da Vinci Xi system follows a standardized approach for positioning the robot over the camera port and targeting of the camera before docking of the remaining robotic ports.

### Positioning and port placement

After insertion of a Foley catheter, the patient is placed in modified lateral flank position with the break of the operating table at the level of the anterior superior iliac crest. The bed may be flexed if desired to permit greater exposure of the abdomen. The anterior abdominal wall is brought toward the edge of the table to allow a greater degree of freedom for the robotic arms without interference from the table. The lower arm of the patient is supported by an arm board and the upper arm is supported by an additional armrest. Both arms may be slightly angled in a cephalad position if more room is needed by the surgical team. The patient is secured to the table with adhesive tape and gel support. The inferior leg is flexed with padding under the knee and ankle. Pillows are placed between the legs to pad the superior leg, which is straight. The area is prepped and draped in the standard fashion.

**Figure 2:**
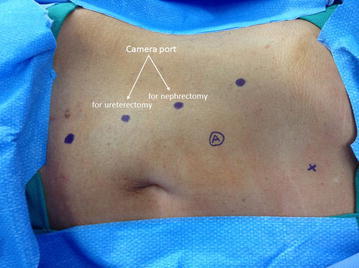
Right sided port placement. *Bullet* 8 mm robotic port, *A* assistant port, *x* 5 mm port.

**Figure 3:**
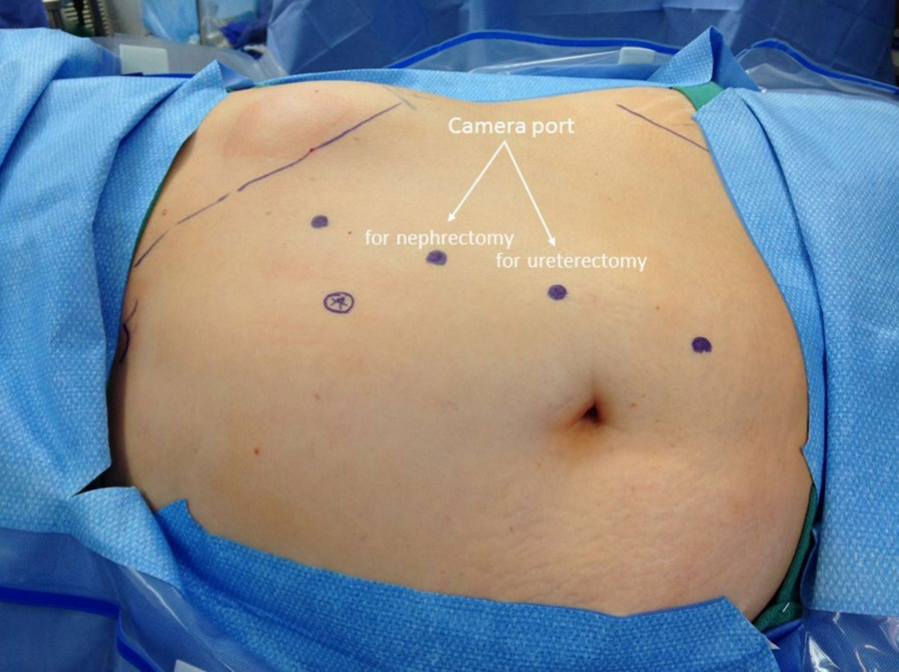
Left sided port placement. *Bullet* 8 mm robotic port, *A* assistant port.

For initial insufflation, a Veress needle technique may be used. We utilize four, 8 mm robotic ports positioned in an oblique straight line starting with a robotic port located two finger breadths below the costal margin just lateral to the rectus abdominis muscle with a minimum distance of 6–8 cm between the ports. The second cephalad port is designated to be the camera port for the initial part (nephrectomy portion) of the procedure. The patient cart is placed on the side of the patient’s back at a right angle with the bed and the robot is brought toward the patient with the target sign of the boom aligned over the camera port. A 30° down lens is used and inserted into the camera port (second cephalad port). The target center is localized to a point between the kidney and ureter. Automatic repositioning of the remaining robotic arms using the targeting feature is performed before connecting to the trocars. All robotic ports are placed in a straight, oblique line (see Figures 2, 3). A 12 mm assistant port is placed closer to the midline and between the two most cephalad robotic ports. A 5 mm port may be placed below the xiphoid for liver retraction for right-sided RALNU. Instruments utilized for RALNU are: monopolar curved scissors (right hand), fenestrated bipolar (left hand), and ProGrasp forceps. Suturing is performed with two large needle drivers.

### Kidney dissection

Dissection is started by incising the white line of Toldt lateral to the colon and mobilizing the colon medially. Medial retraction by the assistant facilitates clearing of the anterior Gerota’s fascia. On the left side, the lienocolic and phrenicocolic ligaments are incised to allow the left colic flexure to fall medially along with the pancreas. On the right side, a Kocher maneuver is performed to mobilize the duodenum and expose the inferior vena cava. Care should be taken to leave the kidney attached laterally to avoid unnecessary mobilization into the operative field. After complete mobilization of the colon, the lower pole of the kidney is identified. Upward traction on Gerota’s fascia and the lower pole tissues will allow identification of the gonadal vein, ureter, and psoas muscle. The ureter is swept laterally and followed proximally to the lower pole of the kidney until identification of the renal hilum. The ureter is dissected from surrounding structures, and a clip is placed on the ureter below the level of the tumor. The renal artery and vein are identified and divided using a vascular stapling device. The plane between the adrenal gland and kidney may be developed and the remaining lateral attachments are divided in order to free the kidney and leave the ureter intact.

### Distal ureterectomy and bladder cuff excision

A unique feature of the da Vinci Xi system is camera port hopping. The camera may be switched to the second caudal trocar at this point in the operation to facilitate visualization of pelvic anatomy for distal ureterectomy and bladder cuff excision. The distal ureter is carefully dissected as it courses over the iliac vessels in order to prevent injury. Ureteral dissection is carried inferiorly to the level of the bladder hiatus and the superior vesicle pedicles can be preserved. The bladder is filled with sterile water and the detrusor muscle is dissected until identification of the bladder mucosa. Bladder cuff excision is performed by incising the mucosa of the bladder in a circumferential manner. An absorbable suture is preplaced at the edge of the cystotomy before completely excising the ureter and bladder cuff in order to maintain traction and control. The bladder defect is then closed in two layers with absorbable, running suture. The integrity of the repair is tested by filling the bladder with sterile water via the Foley catheter. If a leak is identified, additional sutures may be placed until no further extravasation is observed. The nephroureterectomy specimen is placed into a laparoscopic specimen retrieval bag. The renal bed and pelvis are reexamined to ensure hemostasis as the insufflation pressure is lowered to 5 mmHg. A drain is placed and the robot is undocked and all trocars are removed. The specimen may be extracted by making a midline or Gibson incision incorporating the most caudal port.

## Results

A total of 10 patients underwent RALNU without conversion to open surgery. The mean age was 72.1 years (57–86). Six males and four females were included in this study. Mean BMI was 27.6 kg/m^2^ (20–59.6) and mean operating time was 184 min (range 140–300 min), mean estimated blood loss was 121 cc (range 60–300 cc). The average length of hospitalization was 2.4 days. There were no intraoperative complications. One patient developed a pulmonary embolism after being discharged from the hospital. This was subsequently managed with oral anticoagulation therapy. One patient with pre-existing chronic kidney disease (stage IV) required hemodialysis after surgery. Average catheter duration was 7.2 days (2–10 days). The mean follow up period was 130 days (15–210 days) (see Additional file 1: Table S1).

Oncologic results demonstrated pathology consistent with pTa high grade urothelial carcinoma in two patients, pT1 high grade urothelial carcinoma in three patients, pT2 high grade in one patient, and pT3 high grade urothelial carcinoma in four patients. Surgical margins were negative in all patients (see Additional file 1: Table S2).

## Discussion

One of the most challenging aspects of RALNU is removal of the distal ureter and bladder cuff following the nephrectomy portion of the procedure. Positioning and port placement for robotic nephroureterectomy surgery represents a unique challenge as it can affect whether or not the procedure can be performed without additional manipulation of the patient or robotic instrumentation. Although the da Vinci Si surgical system provides a wide range of motion for the robotic arms, rotation of the surgical field from the kidney and retroperitoneum to the pelvis can be challenging. Proper camera and trocar placement is critical to the success of this procedure, and few published papers have described different port placement with or without undocking or repositioning to perform nephroureterectomy using the Si system (Hemal et al. 2011; Rose et al. 2006; Park et al. 2009; Tsivian et al. 2007).

Rose et al. (2006) initially described the use of RALNU. A retroperitoneal approach was utilized with a mean operative time of 183 min, mean blood loss of 75 mL, and no perioperative complications. Other groups have described different techniques for RALNU without repositioning and redocking using the da Vinci Si (Additional file 1: Table S3). Hemal et al. (2011) described a technique of RALNU with bladder cuff excision without intraoperative repositioning using three robotic instruments. The mean operative time was 183 min, mean EBL was 103 mL, and mean hospital stay was 2.7 days. Lee et al. (2013) described a modified paramedian port placement without patient repositioning, port reassignment, or redocking of the robotic arms. The mean operative time was 161 min, mean estimated blood loss was 98.8 mL, and the mean hospital stay was 3 days. Zargar et al. (2014) recently described single step RALNU in 31 patients using two robotic trocars. Mean operative time was 300 min and median hospital stay was 5 days. One patient had a periureteric positive margin with short term recurrence free survival of 77%. Badani et al. (2014) also described a modified port placement technique with single instrument switching during the procedure.

In a population-based study, Trudeau et al. (2014) compared 1,199 patients who underwent laparoscopic nephroureterectomy with 715 patients who underwent RALNU. No significant differences were observed in postoperative or length of stay; however, patients undergoing RALNU were less likely to experience complications compared to patients undergoing laparoscopic nephroureterectomy (p = 0.04). The utilization of RALNU was associated with substantially higher costs compared to the laparoscopic approach (Trudeau et al. 2014).

Lim et al. (2013) reported that mean total operative time could be kept below 200 min by avoiding the need for repositioning the patient for distal ureterectomy and bladder cuff excision. Blood loss with RALNU was also found to be lower than either laparoscopic or open nephroureterectomy series. Oncological outcomes of RALNU remain limited to small, retrospective case series with short follow-up, but these results appeared to be comparable to other approaches.

The da Vinci Xi system offers certain advantages compared to the previous Si platform. The new overhead architecture facilitates anatomical access of different quadrants without repositioning the system. Furthermore, the camera can be placed into any of the robotic ports (port hopping), which provides flexibility for optimally visualizing the renal hilum for nephrectomy and the pelvis for ureterectomy during RALNU. The newly-designed patient clearance joints and longer instrument shafts provide a greater range of motion and reach.

A few disadvantages were encountered with the da Vinci Xi system compared to prior versions of the da Vinci surgical platform. The longer shaft arms of the robotic instrumentation may result in the assistant being further away from patient. Secondly, a visual obturator is currently not available for the camera endoscope. Access into the abdominal cavity with Veress needle pneumoperitoneum followed by blind insertion of a blunt tipped 8 mm trocar or visual access with a 12 mm assistant port trocar is required.

## Conclusion

Robotic-assisted laparoscopic nephroureterectomy using the da Vinci Xi system is a feasible, efficient, and reproducible technique that takes advantage of a novel, in line oblique trocar configuration and port hopping capabilities. As a result, this technique provides efficient visualization and dissection of the kidney and ureter without the need to reposition the patient or robot.

## Additional file

Additional file 1:
**Table S1:** Patient demographics and perioperative characteristics. **Table S2:** Pathological outcomes following RALNU with da Vinci Xi. **Table S3:** Robotic nephroureterectomy series with no repositioning or re-docking approach.
