# Supplementary material for: Operative technique and early experience for robotic-assisted laparoscopic nephroureterectomy (RALNU) using da Vinci Xi
Source: Springerplus. 2015 Jun 27;4:298. doi: 10.1186/s40064-015-1076-6 (PMC4483175; doi:10.1186/s40064-015-1076-6)
Supplement: Additional file 1: — Table S1: Patient demographics and perioperative characteristics. Table S2: Pathological outcomes following RALNU with da Vinci Xi. Table S3: Robotic nephroureterectomy series with no repositioning or re-docking approach. [file 40064_2015_1076_MOESM1_ESM.docx]

**Table S1.** Patient demographics and perioperative characteristics.

| **Age** | **Sex** | **BMI** | **Side** | **CT scan** | **O-time mn** | **C-time mn** | **EBL** | **Ocx** | **POCx** | **LS** | **Cath** |
| --- | --- | --- | --- | --- | --- | --- | --- | --- | --- | --- | --- |
| 78 | M | 21.6 | R | R renal pelvis tumor 3.9cm | 166 | 85 | 300 | no | no | 2 | 2 |
| 57 | M | 26.8 | L | L distal enhancing ureteral lesion 3 x 1.2 m | 155 | 90 | 150 | no | no | 2 | 7 |
| 79 | M | 27.1 | L | L upper pelvicalyceal system filling defect | 176 | 120 | 100 | no | no | 2 | 10 |
| 57 | F | 59.6 | R | R renal pelvis soft tissue density mass 3.6 x 2.4cm | 300 | 160 | 100 | no | no | 2 | 10 |
| 81 | M | 21.6 | R | R lower ureter tumor | 140 | 70 | 100 | no | HD | 4 | 3 |
| 72 | F | 22.6 | L | L renal pelvis tumor 2 cm | 156 | 60 | 100 | no | PE | 3 | 3 |
| 61 | M | 26.3 | R | R distal ureter | 208 | 140 | 100 | no | no | 2 | 10 |
| 86 | M | 26.8 | R | R renal pelvis lesion | 204 | 139 | 100 | no | no | 2 | 10 |
| 75 | F | 20 | R | R distal ureteral tumor 3cm | 189 | 125 | 100 | no | no | 2 | 9 |
| 75 | F | 23.9 | L | L renal pelvis lesion 2cm | 146 | 66 | 60 | no | no | 3 | 8 |

BMI: Body Mass Index, R: Right, L: Left, CT scan: Preoperative CT findings, O-time: operative time in minutes, C-time: console time in minutes, EBL: estimated blood loss, OCx: intraoperative complications, POCx: post-operative complications, LS: length of hospital stay in days, Cath: catheter duration in days, HD: hemodialysis .

**Table S2.** Pathological outcomes following RALNU with da Vinci Xi.

| **Preop pathology** | **Final path** | **Location** | **SM** |
| --- | --- | --- | --- |
| Not available | pT1Nx HGUC | pelvicalyceal system | Negative |
| Low grade papillary UC | pT1N0 HGUC | renal pelvis and ureter | Negative |
| Wash cytology: left kidney positive | pTaNx HGUC | renal pelvis | Negative |
| Right kidney cytology: atypical cells | pTaNx HGUC | renal pelvis | Negative |
| Invasive HGUC at least T1 | pT3Nx HGUC + Cis | lower ureter | Negative |
| Papillary noninvasive HGUC | pT3Nx HGUC | renal pelvis | Negative |
| Atypical cytology | pT3N0 HGUC | lower ureter | Negative |
| Low grade UC | pT1N0 HGUC | renal pelvis | Negative |
| Mixed high grade and low grade UC | pT2N0 HGUC | renal pelvis and ureter | Negative |
| Left renal fluid cytology positive for UC | pT3N0 HGUC | renal pelvis and ureter | Negative |

Cis: carcinoma in situ, Final path: final pathology, HGUC: High Grade Urothelial Carcinoma, Preop path: preoperative pathology/cytology, SM: Surgical Margin , UC: urothelial carcinoma.

**Table S3**. Robotic nephroureterectomy series with no repositioning or re-docking approach.

| **Series** | **# of cases** | **Operative time (min)** | **EBL (cc)** | **LOS (days)** | **Surgical technique** |
| --- | --- | --- | --- | --- | --- |
| **Lee et al**. (2013) | 20 | 161 | 99 | 3 | Modified paramedian line |
| **Hemal et al**. (2011) | 15 | 184 | 103 | 3 | Ports lateral to rectus margin + 4^th^ arm cranial to the iliac crest |
| **Zargar et al**. (2014) | 31 | 300 | 200 | 5 | Straight line,lateral to rectus, no 4th arm |
| **Park et al.** (2009) | 5 | 193 | 270 | 8.4 | Hybrid port |
| **Badani et al.** (2014) | 26 | 230 | 66 | 2 | Modified port placement, single instrument switch |
| **Present series** (2015) | 10 | 184 | 121 | 2.4 | Da Vinci Xi, camera port hopping, oblique in-line port configuration |
